# Impact of Thermochemical Treatments on Electrical Conductivity of Donor-Doped Strontium Titanate Sr(Ln)TiO_3_ Ceramics

**DOI:** 10.3390/ma17153876

**Published:** 2024-08-05

**Authors:** Aleksandr Bamburov, Ekaterina Kravchenko, Aleksey A. Yaremchenko

**Affiliations:** CICECO—Aveiro Institute of Materials, Department of Materials and Ceramic Engineering, University of Aveiro, 3810-193 Aveiro, Portugal; che.kravchenko@gmail.com

**Keywords:** perovskite, titanate, SrTiO_3_, donor doping, electrical conductivity, SOFC, SOEC, redox stability, grain boundary

## Abstract

The remarkable stability, suitable thermomechanical characteristics, and acceptable electrical properties of donor-doped strontium titanates make them attractive materials for fuel electrodes, interconnects, and supports of solid oxide fuel and electrolysis cells (SOFC/SOEC). The present study addresses the impact of processing and thermochemical treatment conditions on the electrical conductivity of SrTiO_3_-derived ceramics with moderate acceptor-type substitution in a strontium sublattice. A-site-deficient Sr_0.85_La_0.10_TiO_3−δ_ and cation-stoichiometric Sr_0.85_Pr_0.15_TiO_3+δ_ ceramics with varying microstructures and levels of reduction have been prepared and characterized by XRD, SEM, TGA, and electrical conductivity measurements under reducing conditions. The analysis of the collected data suggested that the reduction process of dense donor-doped SrTiO_3_ ceramics is limited by sluggish oxygen diffusion in the crystal lattice even at temperatures as high as 1300 °C. A higher degree of reduction and higher electrical conductivity can be obtained for porous structures under similar thermochemical treatment conditions. Metallic-like conductivity in dense reduced Sr_0.85_La_0.10_TiO_3−δ_ corresponds to the state quenched from the processing temperature and is proportional to the concentration of Ti^3+^ in the lattice. Due to poor oxygen diffusivity in the bulk, dense Sr_0.85_La_0.10_TiO_3−δ_ ceramics remain redox inactive and maintain a high level of conductivity under reducing conditions at temperatures below 1000 °C. While the behavior and properties of dense reduced Sr_0.85_Pr_0.15_TiO_3+δ_ ceramics with a large grain size (10–40 µm) were found to be similar, decreasing grain size down to 1–3 µm results in an increasing role of resistive grain boundaries which, regardless of the degree of reduction, determine the semiconducting behavior and lower total electrical conductivity of fine-grained Sr_0.85_Pr_0.15_TiO_3+δ_ ceramics. Oxidized porous Sr_0.85_Pr_0.15_TiO_3+δ_ ceramics exhibit faster kinetics of reduction compared to the Sr_0.85_La_0.10_TiO_3−δ_ counterpart at temperatures below 1000 °C, whereas equilibration kinetics of porous Sr_0.85_La_0.10_TiO_3−δ_ structures can be facilitated by reductive pre-treatments at elevated temperatures.

## 1. Introduction

Solid oxide cells are an advanced electrochemical energy conversion and storage technology that can be used for generating electrical energy in fuel cell mode (solid oxide fuel cell, SOFC) [1], converting excess green renewable power into fuels or chemicals in electrolysis mode (solid oxide electrolysis cell, SOEC) [2], and balancing electrical grids in a reversible regime (reversible solid oxide cell, rSOC) [3]. The development of solid oxide cell technology faces significant material challenges, including the requirements for high-temperature stability, compatibility of different cell components, and long-term durability under operation conditions.

Remarkable stability in a wide range of thermochemical conditions, moderate thermal expansion, and acceptable electrical conductivity (≥10 S/cm) attracted significant interest to donor-doped SrTiO_3_ ceramics as prospective components for SOFC anodes [4,5,6,7,8]. Resistance to carbon deposition and sulfur poisoning makes these materials promising candidates for anodes operating in harsh environments—in SOFCs fueled by natural gas or biogas [9,10,11,12] and direct-carbon SOFCs [13,14,15]. In the last decade, donor-doped strontium titanates were also explored as prospective materials for SOEC cathodes [16,17,18,19], interconnects and current collectors for SOFC/SOEC [20,21,22,23], and supports for thin-electrolyte cells [24,25,26,27].

**Figure 1:**
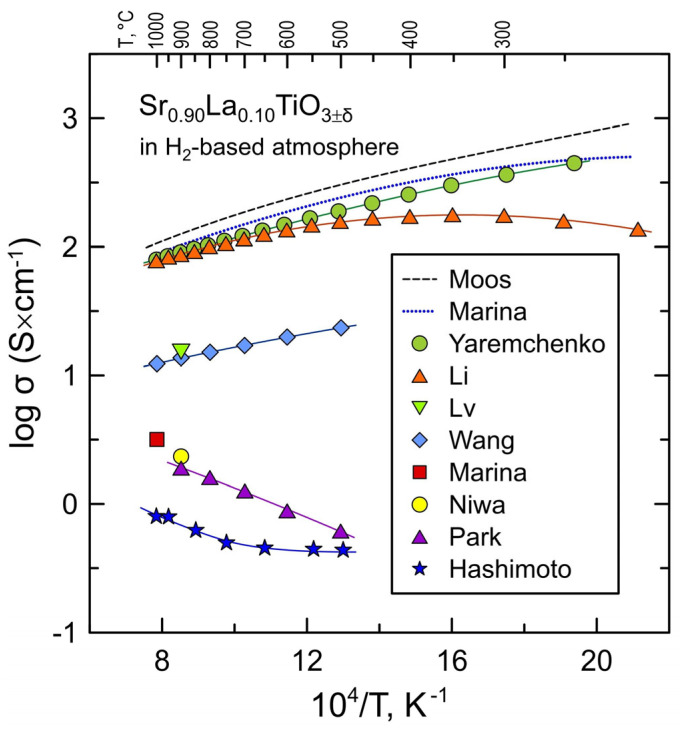
Comparison of literature data on the electrical conductivity of Sr_0.90_La_0.10_TiO_3±δ_ ceramics in reducing H_2_-based atmospheres. Sources: Moos [37], Marina [36], Yaremchenko [35], Li [38], Lv [39], Wang [40], Niwa [41], Park [42], and Hashimoto [43]. See Table 1 for the experimental details.

An acceptable level of electrical conductivity is one of the main requirements for all mentioned applications in solid oxide cells. The high electrical conductivity of components is a prerequisite to minimize the ohmic losses during SOFC/SOEC operation. In addition, insufficient electrical conductivity of electrode material may be a factor limiting its electrochemical performance [28,29]. The target electrical conductivity for electrode materials is often set to be around 100 S/cm in a dense form (and one order of magnitude lower for porous structures) [30]. Even higher electrical conductivity is preferable for interconnect materials to satisfy the ASR (area-specific resistance) requirement of <10 mΩ × cm^2^ [31]. While undoped SrTiO_3_ is a wide-bandgap semiconductor with a comparatively low electrical conductivity [32,33], donor-type substitutions into either the Sr^2+^ sublattice (e.g., by rare-earth cations Ln^3+^) or into the Ti^4+^ sublattice (e.g., by Nb^5+^ or Ta^5+^) promote a significant increase in the n-type electronic conductivity under reducing conditions [5,6,7,8,34,35,36]. At the same time, the literature reports provide very non-uniform data on electrical conductivity even for the ceramics of the same composition which is caused by diverse conditions used for fabrication and thermochemical treatments of ceramic samples and their electrical characterization. As an example, Figure 1 and Table 1 summarize the literature data [35,36,37,38,39,40,41,42,43] on the electrical conductivity of Sr_0.90_La_0.10_TiO_3±δ_ ceramics measured under reducing conditions, in hydrogen-based atmospheres. The plot shows that, depending on ceramic processing conditions, the reported values of electrical conductivity may vary within 2–2.5 orders of magnitude under reducing conditions and temperatures relevant for SOFC/SOEC operation.

The present work aims to explore the effects of thermochemical processing conditions and microstructure on the electrical conductivity of two SrTiO_3_-derived compositions with moderate levels of donor-type substitution in the strontium sublattice but different defect chemistry: Sr_0.85_La_0.10_TiO_3−δ_ and Sr_0.85_Pr_0.15_TiO_3±δ_. A-site-deficient Sr_0.85_La_0.10_TiO_3−δ_ is a representative of the Sr_1−1.5*x*_Ln*_x_*TiO_3−δ_ series. Donor-type substitution in these series of titanates under oxidizing conditions is charge-compensated by strontium vacancies:

(1) $$\left[Ln_{Sr}^{3+}]=0.5[V_{Sr}\right]\;\mathrm{or}\;[Ln_{Sr}^{\cdot }]=0.5[V_{Sr}^{\prime \prime }],$$

The reduction process involves partial reduction of titanium cations from the 4+ to 3+ oxidation state accompanied by the formation of vacancies in the oxygen sublattice:

(2) $$Sr_{1-1.5x}Ln_{x}Ti^{4+}O_{3}\overset{red}{\to }Sr_{1-1.5x}Ln_{x}Ti_{1-2\delta }^{4+}Ti_{2\delta }^{3+}O_{3-\delta },$$

Nominally cation-stoichiometric Sr_0.85_Pr_0.15_TiO_3±δ_ belongs to the Sr_1−x_Ln_x_TiO_3+δ_ series with the donor dopant charge-compensated by oxygen excess in the structure. In this case, defect chemistry may involve either (i) simultaneous formation of Sr vacancies and extended defects—SrO shear planes characteristic of layered Ruddlesden–Popper structures or (ii) oxygen-rich $Ln_{Sr}^{3+}\cdot \cdot \cdot O_{i}^{2-}$ defect clusters [6,44,45,46]. When Sr vacancies and SrO shear plains are prevailing defects, the formula of oxidized oxide can be expressed as follows:

Sr_1−*x*_Ln_*x*_TiO_3+δ_ ≡ Sr_1−1.5x_Ln_*x*_TiO_3_∗(SrO)_0.5x_, (3)

and the full reduction at elevated temperatures may also involve the dissolution of extended defects:

(4) $$Sr_{1-1.5x}Ln_{x}Ti^{4+}O_{3}*{\left(SrO\right)}_{0.5x}\overset{red}{\to }Sr_{1-x}Ln_{x}Ti_{1-x-2\delta }^{4+}Ti_{x+2\delta }^{3+}O_{3-\delta },$$

At lower temperatures, when the cation diffusivity is negligible, the reduction should still be limited to a process similar to Equation (2).

In this study, powders of both compositions were synthesized in a similar manner. Ceramic samples with varying morphologies and degrees of reduction were prepared under different thermochemical processing conditions. Their electrical conductivity was examined under reducing conditions typical for the fuel electrodes in solid oxide cells. The results aim to identify the factors that may affect the electrical properties of strontium titanate-derived ceramics and provide guidelines for optimizing donor-doped SrTiO_3_-based components for solid oxide cell applications.

## 2. Materials and Methods

Sr_0.85_La_0.10_TiO_3−δ_ (S85L10) and Sr_0.85_Pr_0.15_TiO_3±δ_ (S85P15) titanates were synthesized by the solid-state reaction method. SrCO_3_ (≥99.9%, Sigma-Aldrich,), TiO_2_ (anatase, 99.8%, Sigma-Aldrich,), La_2_O_3_ (99.9%, Sigma-Aldrich), and Pr_6_O_11_ (99.9%, Sigma-Aldrich) were used as starting reagents. Before weighing, oxides were calcined at 1000 °C for 2 h in air to remove adsorbates. The mixtures of precursors were calcined at 900–1300 °C, increasing the temperature in a stepwise manner with a step of 100 °C, duration of 5 h at each temperature, and regrinding in a mortar between the steps. Then, the synthesized powders were ball-milled (Retsch S1 planetary mill (Retsch GmbH, Haan, Germany), nylon containers with tetragonal zirconia balls) with ethanol for 4 h at 150 rpm, dried, and compacted into disk-shaped pellets (Ø 18 mm, thickness of ~3 mm). The green compacts were either sintered in air with subsequent reductive treatment in the flow of 10%H_2_-N_2_ gas mixture or sintered directly in a reducing 10%H_2_-N_2_ atmosphere. The conditions of sintering and thermochemical treatments as well as corresponding notations of the samples are listed in Table 2.

The prepared ceramic pellets were polished and cut into rectangular bars (approximate dimensions 2.5 mm × 2.5 mm × 12 mm) for electrical measurements. The experimental density was calculated from the mass and geometric dimensions of polished samples. Powdered samples for X-ray diffraction (XRD) and thermogravimetric analysis (TGA) were prepared by grinding sintered pellets in a mortar.

XRD patterns were recorded at room temperature using a PANalytical X’Pert PRO MRD diffractometer (PANalytical, Almelo, The Netherlands, CuKα radiation) in the range 2θ = 20–80°. Unit cell parameters were calculated using FullProf software (version March 2021) (profile match method). Microstructural characterization was conducted by scanning electron microscopy (SEM, Hitachi SU-70 (Hitachi, Tokyo, Japan)). The grain sizes were estimated by analyzing the collected SEM images of fractured ceramics using ImageJ 1.54 software [47,48]. Thermogravimetric analysis (TGA, Setaram SetSys 16/18 instrument (Setaram, Caluire, France), sensitivity 0.4 µg, initial sample weight 0.4–0.5 g) was carried out in flowing air or 10%H_2_-N_2_ mixture at 25–1100 °C with a constant heating/cooling rate of 2 °C/min.

The electrical conductivity was determined by the 4-probe DC technique as a function of temperature at 300–1000 °C in a 10%H_2_-N_2_ atmosphere and function of oxygen partial pressure at 900 °C in a H_2_-H_2_O-N_2_ atmosphere. The measurements were performed with a stepwise change in temperature (cooling regime) or p(O_2_) (in the regime of increasing oxygen partial pressure) and equilibration for 1–2 h at each step. Variations in the conductivity of ceramic samples on isothermal redox cycling between air and 10%H_2_-N_2_ atmospheres were measured by pseudo-4-probe AC impedance spectroscopy (Agilent 4284A precision LCR meter (Agilent, Santa Rosa, CA, USA)) using bar-shaped samples with porous Pt electrodes applied onto the end faces of the bars. The flow rates of gases in the course of electrical measurements were controlled using Bronkhorst mass-flow controllers (Ruurlo, The Netherlands).

Oxygen partial pressure p(O_2_) in the flowing gas mixtures during the preparation and characterization of ceramic materials was monitored using 8 mol.% yttria-stabilized zirconia (YSZ) solid-electrolyte sensors. Oxygen partial pressure in the H_2_-H_2_O-N_2_ atmosphere was defined by the p(H_2_O)/p(H_2_) ratio and the equilibrium constant of the hydrogen oxidation reaction; the total fraction of H_2_ + H_2_O in nitrogen corresponded to ~10 vol.%. In the case of the 10%H_2_-N_2_ mixture, the p(O_2_) was determined by residual water content and by leaks of oxygen from the ambient atmosphere; the representative value of p(O_2_) in the nominal 10%H_2_-N_2_ atmosphere corresponded to ~10^−21^ atm at 900 °C.

## 3. Results and Discussion

### 3.1. Crystal Structure and Microstructure of Prepared Ceramics

**Figure 2:**
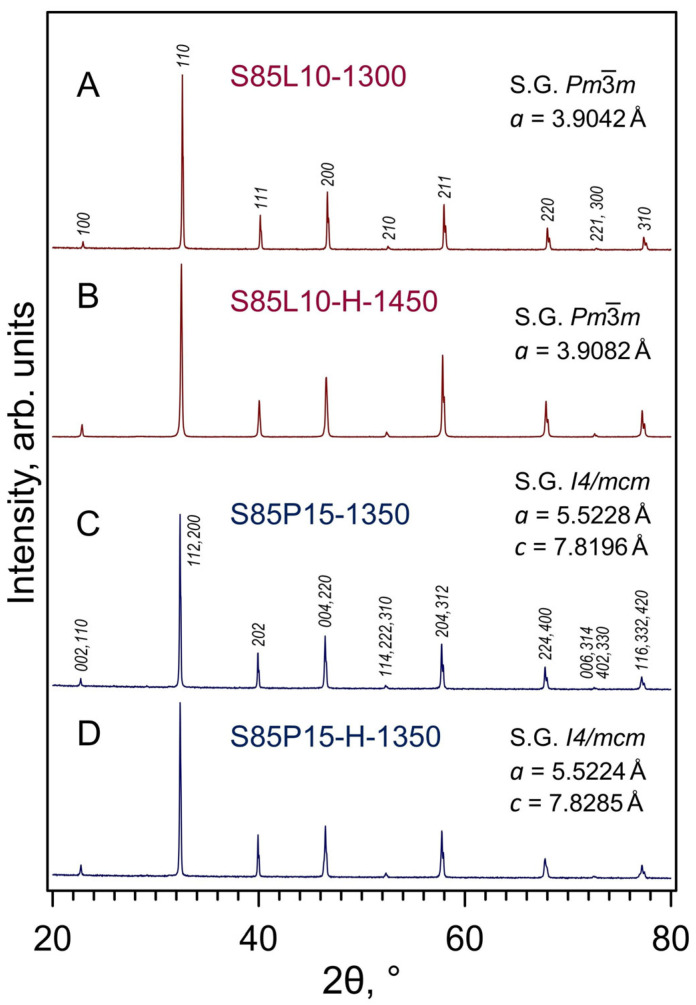
Examples of XRD patterns of S85L10 and S85P15 samples. Note that S85L10-1300 indicates the powder as synthesized in air at 1300 °C, and S85P15-1350 corresponds to the ceramic sample sintered in air at 1350 °C. The notations of other samples are listed in Table 2.

As-synthesized powder of S85L10 was phase-pure after calcinations at 1300 °C and had a cubic perovskite-type structure (space group $Pm\bar{3}m$) (Figure 2A), in agreement with the literature data [35,49]. The cubic perovskite structure was maintained after sintering and thermochemical treatment under different conditions (e.g., Figure 2B); no evidence of phase impurities could be detected by XRD.

**Figure 3:**
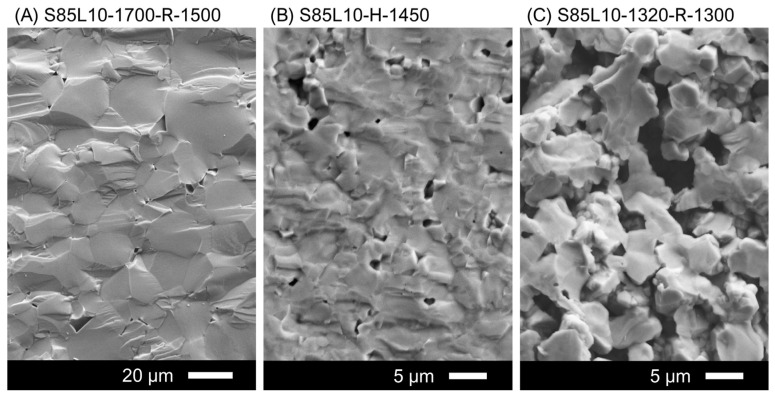
SEM images of fractured cross-sections of S85L10 ceramics prepared under different conditions.

The sintering of S85L10 in air at 1700 °C promoted grain growth, up to 5–31 µm, and densification. The microstructure was preserved after subsequent reductive treatment at 1300–1500 °C in a 10%H_2_-N_2_ atmosphere (Figure 3A); the samples were dense with a residual closed porosity of 4–5 vol.% (Table 2). Direct sintering in a reducing 10%H_2_-N_2_ gas mixture at 1450 °C yielded a different microstructure (Figure 3B). The temperature was not high enough to induce significant grain growth; the maximum grain size of prepared ceramics was limited to 6.8 µm (Table 2). Still, S85L10-H-1450 ceramics had a relative density of 91% with a comparatively low fraction of closed porosity. On the other hand, pre-sintering and reduction at lower temperatures, 1300–1320 °C, produced porous S85L10-1320-R-1300 ceramic samples with a similar grain size but with a volume fraction of interconnected pores of 30% (Table 2). The individual grains were mostly combined into larger agglomerates (Figure 3C).

XRD analysis of S85P15 samples sintered at 1350 °C either in air or in a 10%H_2_-N_2_ atmosphere confirmed the formation of single-phase perovskite ceramics (Figure 2C,D). The shape of selected XRD reflections (at 2θ = 46.3–46.7° and 77.0–77.5°) implied, however, that the symmetry of the crystal lattice is lower than cubic. The XRD patterns of S85P15 were successfully indexed in the tetragonal *I*4/*mcm* space group (Figure 2C). This is in agreement with the literature data showing that increasing praseodymium content in the Sr_1−*x*_Pr*_x_*TiO_3±δ_ series induces a transition from cubic to tetragonal structure at *x*~0.10 [34,50,51]. The phase-pure tetragonally distorted perovskite structure was preserved after thermochemical treatments of S85P15 samples at higher temperatures (1500–1700 °C).

**Figure 4:**
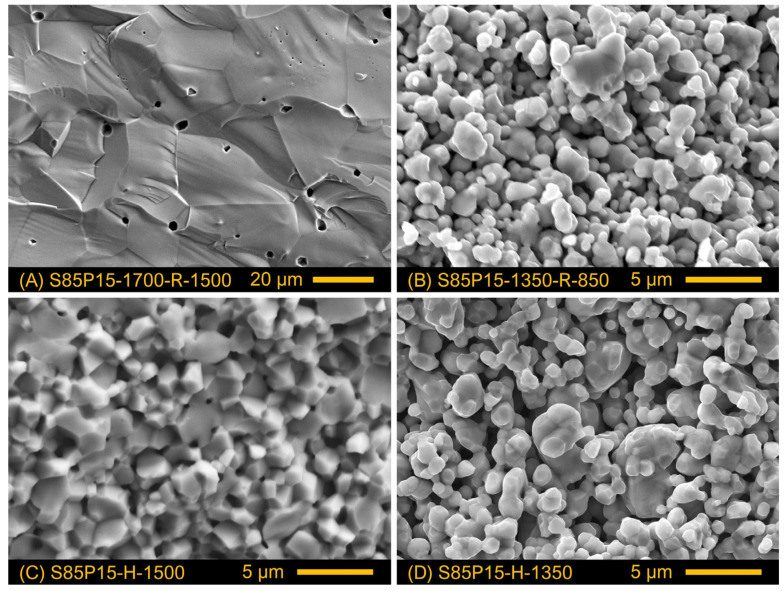
SEM images of fractured cross-sections of S85P15 ceramics prepared under different conditions.

Similar to S85L10 ceramics, the microstructure of prepared S85P15 samples was mainly defined by the sintering temperature. Sintering in air at an elevated temperature, 1700 °C, promoted densification and substantial grain growth, with grain sizes varying in the range between 10 and 38 µm. The microstructure was not altered by subsequent reduction at 1500 °C (Figure 4A); the estimated relative density of the samples was 90% (Table 2). Direct sintering in a reducing atmosphere at 1500 °C yielded ceramic samples with a similar relative density (Table 2) but substantially smaller grain size in the range of 0.8–3.5 µm (Figure 4C). A similar grain size was obtained after sintering at a lower temperature, 1350 °C, in either an oxidizing or reducing atmosphere (Table 2 and Figure 4B,D), although the produced ceramic samples were porous with the estimated fraction of open porosity corresponding to 27–35 vol.%. One should note that the relative density of prepared S85P15 ceramics may be somewhat underestimated. While all prepared S85P15 samples were oxygen-overstoichiometric (as discussed below) with excess oxygen and a fraction of cations accommodated presumably as extended defects, the estimations of theoretical density based on the lattice parameters did not take into account the presence of such defects and were done assuming the nominal elemental composition.

### 3.2. Electrical Conductivity of S85L10 Ceramics

**Figure 5:**
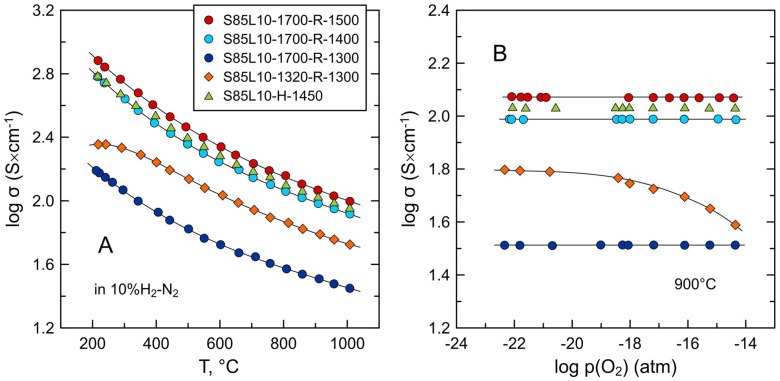
Electrical conductivity of S85L10 ceramics as a function of (**A**) temperature in 10%H_2_-N_2_ atmosphere and (**B**) oxygen partial pressure under reducing conditions at 900 °C.

Figure 5A shows the temperature dependence of the electrical conductivity of S85L10 ceramics prepared under different conditions. Dense samples (i.e., with relative density > 90%) demonstrate metallic-like behavior in the studied temperature range between 200 and 1000 °C with the conductivity decreasing on heating. The level of electrical conductivity correlates with the temperature of thermochemical treatment under reducing conditions. The values of conductivity at 600–1000 °C increase nearly linearly with increasing processing temperature from 1400 to 1500 °C but drop for the S85L10-1700-R-1300 sample reduced at 1300 °C. The conductivity of porous S85L10-1320-R-1300 ceramics reduced at 1300 °C also shows metallic-like temperature dependence above 300 °C but tends to semiconducting behavior at low temperatures. At the same time, the conductivity of the porous S85L10-1320-R-1300 sample exceeds that of the dense S85L10-1700-R-1300 sample by ~2 times despite the identical reduction conditions.

These results imply that two main factors define the level of electrical conductivity in prepared S85L10 ceramics: the temperature of thermochemical treatment under reducing conditions and the morphology of the samples (porous or dense). The reduction process in S85L10 involves the formation of two types of point defects—Ti^3+^ cations and oxygen vacancies:

(5) $$2Ti_{Ti}^{\times }+O_{O}^{\times }\;\;\overset{red}{\underset{ox}{⇄}}\;2Ti_{Ti}^{\prime }+V_{O}^{\cdot \cdot }+0.5O_{2},$$

The kinetics of reduction are expected to be governed by the diffusion of oxygen vacancies from the surface into the bulk of ceramics (or oxygen ions from the interior to the surface). The experimental data on oxygen diffusivity in donor-doped titanates are scarce. Kiessling et al. [52] reported that oxygen tracer diffusion coefficients *D* in single-crystal lanthanum-doped SrTiO_3_ are in the range between 2 × 10^−15^ and 1 × 10^−13^ cm^2^/min at 700–900 °C. Estimations made by the approximation of these data to higher temperatures suggest that the spatial scale of the oxygen diffusion process, ~$\sqrt{2Dt}$, corresponds to ~6.7 µm at 1300 °C and ~13.3 µm at 1500 °C in a timescale *t* of 10 h. This indicates that the reduction kinetics of ceramics with grain sizes up to 31 µm (Table 2) may be limited by sluggish diffusion even at the grain size scale (even if diffusion along the grain boundaries is substantially faster than in the grain interior). Analysis of electrical conductivity data seems to imply that, regardless of the grain size, the samples are likely to attain a state close to equilibrium in the course of processing at 1400–1500 °C in a reducing atmosphere. At the same time, the reduction of dense S85L10-1700-R-1300 is not accomplished at 1300 °C due to slower diffusion kinetics and insufficient time. This should result in a combination of reduced shells and oxidized cores at different scales, creating radial gradients of redox states from the surface to the interior of individual grains at the microscale and throughout the entire dense ceramic sample at the macroscale. On the contrary, comparatively small grain size and porous morphology with easy access of the gas phase to the individual grains ensures faster reduction and, as a result, higher conductivity of S85L10-1320-R-1300 reduced at the same temperature.

Considering the sluggish kinetics of equilibration even at 1300 °C, the state of dense S85L10 ceramics characterized at temperatures ≤ 1000 °C should be regarded as quenched from the processing temperatures. This is supported by the results of the measurements of electrical conductivity as a function of oxygen partial pressure (Figure 5B). All dense S85L10 samples showed p(O_2_)-independent conductivity at 900 °C in the studied oxygen partial pressure range. This implies that possible oxygen exchange processes are limited to the thin surface layer while electrical properties are determined by the quenched reduced state of the sample interior. On the contrary, porous S85L10-1320-R-1300 ceramics were found to be redox-active (Figure 5B). Increasing oxygen partial pressure results in a decrease in conductivity of this sample due to a decrease in the concentration of n-type electronic charge carriers according to Equation (5) which can rewritten as follows:

(6) $$\;2e^{\prime }+V_{O}^{\cdot \cdot }+0.5O_{2}\;\overset{ox}{\underset{red}{⇄}}\;O_{O}^{\times },$$

The concentration of n-type charge carriers is equivalent to the concentration of Ti^3+^ cations and is interrelated with the oxygen deficiency through the electroneutrality condition:

*n* = [Ti^3+^] = 2δ, (7)

**Figure 6:**
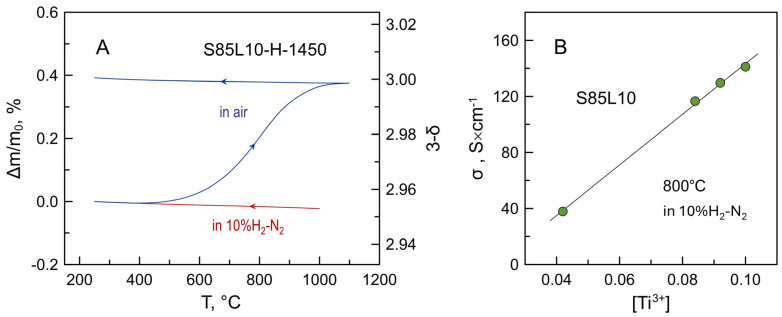
(**A**) Example of thermogravimetric data recorded for powdered S85L10 (Sr_0.85_La_0.10_TiO_3−δ_) ceramics on cooling in 10%H_2_-N_2_ atmosphere and subsequent heating/cooling cycle in air. (**B**) Electrical conductivity of dense S85L10 ceramics vs. fraction of Ti^3+^ cations in the titanium sublattice.

These values can be estimated from the results of thermogravimetric studies. Figure 6A shows an example of thermogravimetric data collected for powdered S85L10-H-1450 ceramics. The variations in the sample weight were negligible on cooling in a 10%H_2_-N_2_ atmosphere. On heating in air, the oxidation started above 400 °C and was accomplished when the temperature approached 1100 °C. The sample was kept at 1100 °C for 5 h to ensure that the sample weight was constant. The weight changes were insignificant on subsequent cooling. Assuming that all titanium cations in the oxidized sample are in the 4+ oxidation state, one may estimate oxygen nonstoichiometry and the fraction of Ti^3+^ cations in the original reduced ceramics from the weight gain on oxidation (Figure 6A).

Figure 6B shows the electrical conductivity of dense S85L10 ceramics at 800 °C plotted vs. the estimated fraction of Ti^3+^ (or concentration of electronic charge carriers). The data show a nearly linear relationship as expected from the following definition:

σ = *e* µ*_e_ n*, (8)

where *e* is the elementary charge, and µ*_e_* is electron mobility. Interestingly, the linear dependence is observed even though the estimated concentration of electronic charge carriers in the dense S85L10-1700-R-1300 is not uniform (due to non-uniform reduction) and the obtained value is rather average for the sample.

**Figure 7:**
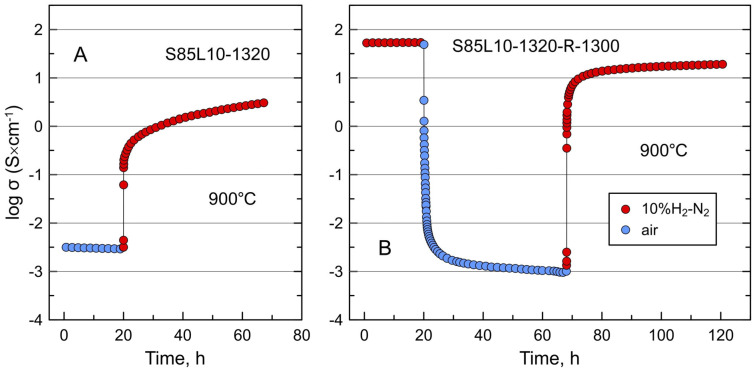
Relaxation of electrical conductivity of (**A**) oxidized S85L10-1320 ceramics on reduction and (**B**) reduced S85L10-1320-R-1300 ceramics on redox cycling at 900 °C.

Figure 7 compares the relaxation of electrical conductivity of two samples with expectedly identical porous morphology on redox cycling at 900 °C. S85L10-1320 is a sample sintered in air at 1320 °C, while S85L10-1320-R-1300 is a similar sample that was reduced in 10%H_2_-N_2_ atmosphere at 1300 °C. The porous S85L10-1320 sample showed a low electrical conductivity in air, 3 × 10^−3^ S/cm at 900 °C (Figure 7A). Switching to reducing 10%H_2_-N_2_ atmosphere results in a rapid rise in conductivity by approximately two orders of magnitude followed by a slower further increase with time. This can be interpreted as a fast initial reduction of the surface of individual grains and agglomerated particles followed by a sluggish propagation of the reduction front into the interior of grains. After more than 25 h of reduction, the conductivity reaches 3.0 S/cm and continues to grow, with no evident tendency to a constant equilibrium value. The reduced S85L10-1320-R-1300 sample exhibited an initial conductivity of 54 S/cm in a reducing atmosphere which drops by nearly five orders of magnitude, down to 1 × 10^−3^ S/cm, on oxidation in air at 900 °C (Figure 7B). Switching back to a reducing atmosphere induces a sharp increase in the conductivity by around four orders of magnitude followed by a slower further drift to higher values. After 50 h of re-reduction at 900 °C, the conductivity reached 19 S/cm and continued to increase slowly, but it was evident that it would not return to the initial value. These observations confirm the slow kinetics of reduction of donor-doped titanates at temperatures below 1000 °C reported earlier [34,39,40,44,53,54]. It seems also that, similar to dense ceramics, the as-processed porous S85L10-1320-R-1300 sample remains in a state partly quenched from processing temperature, and the conductivity value after the oxidation–reduction cycle is closer to equilibrium. Preliminary reduction at elevated temperatures induces faster kinetics of equilibration on oxidation–reduction compared to original oxidized ceramics despite a similar porous morphology and microstructure. The reasons for that are not evident from the collected experimental data.

### 3.3. Electrical Conductivity of S85P15 Ceramics

**Figure 8:**
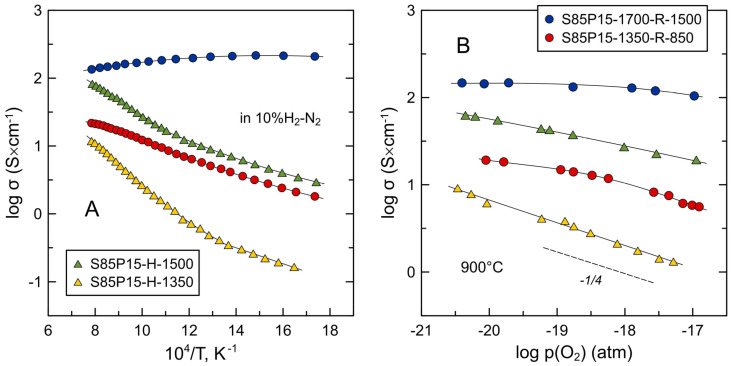
Electrical conductivity of S85P15 ceramics as a function of (**A**) temperature in 10%H_2_-N_2_ atmosphere and (**B**) oxygen partial pressure under reducing conditions at 900 °C.

Figure 8A compares the electrical conductivity of S85P15 ceramics processed under different conditions. The impact of processing conditions on the behavior of this “cation-stoichiometric” oxide is more complex compared to the A-site-deficient S85L10. Only dense S85P15-1700-R-1500 ceramics exhibited metallic-like conductivity at elevated temperatures but showed a transition to semiconducting behavior below 400 °C. In the high-temperature range, above 680 °C, the conductivity of this sample exceeds that of the S85L10 counterpart. Despite the similar relative density, the S85P15-H-1500 sample sintered directly in a reducing atmosphere showed noticeably lower conductivity; due to the thermally activated character of conductivity, the difference substantially increases on cooling. Compared to the S85P15-H-1500 sample, S85P15-H-1350 ceramics exhibited approximately one order of magnitude lower electrical conductivity in the entire studied temperature range. The shape of conductivity curves is similar for both samples in Arrhenius coordinates, with a change in slope at 550–600 °C. The measured conductivity of the porous S85P15-1350-R-850 sample reduced at a lower temperature, 850 °C, exceeded that of S85P15-H-1350 ceramics. The activation energy E_A_ of electrical conductivity of this sample remained nearly constant in the entire studied temperature range and was similar to the corresponding E_A_ values for the S85P15-H ceramics at lower temperatures (Figure 8A).

**Figure 9:**
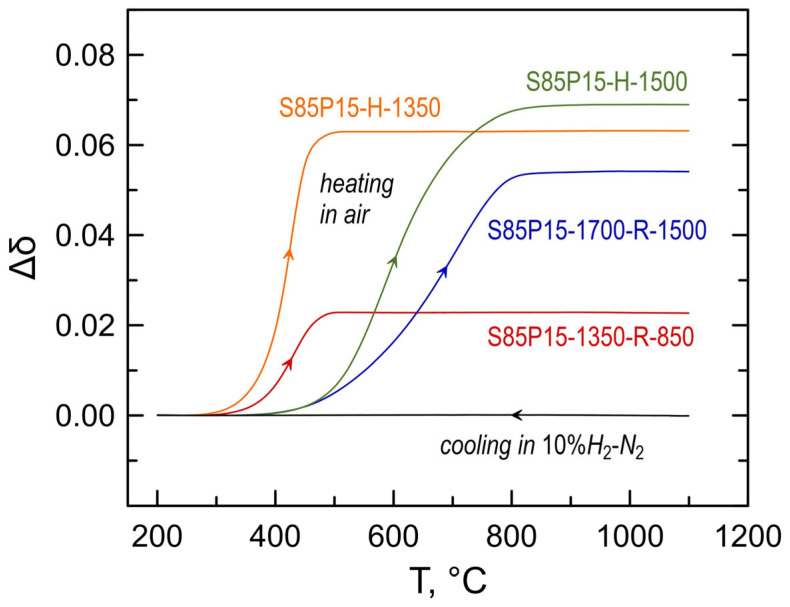
Changes in oxygen nonstoichiometry of S85P15 (Sr_0.85_Pr_0.15_TiO_3+δ_) ceramics on oxidation in air estimated from the thermogravimetric data.

In order to examine relationships between the concentration of electronic charge carriers and the level of electrical conductivity, the degree of reduction of S85P15 ceramics prepared under different conditions was evaluated by TGA in a similar manner as it was conducted for S85L10 samples. Figure 9 shows the estimated relative changes in oxygen nonstoichiometry caused by oxidation on heating in air. The S85P15-1350-R-850 sample was preliminarily heated to 1100 °C in the 10%H_2_-N_2_ atmosphere; this promoted a stronger reduction compared to the initial sample reduced at 850 °C but better describes the state of the sample during electrical conductivity measurements (Figure 8A). Another note is that, in the case of S85P15 ceramics, titanium is not the only cation that may undergo a change in oxidation state. The gain in the oxygen content may be partially contributed by Pr^3+^ → Pr^4+^ oxidation. Earlier, XPS studies revealed that praseodymium cations in the bulk of oxidized Sr_0.90−*x*_Pr_0.10_TiO_3±δ_ (*x* = 0 and 0.05) ceramics have a preference for the 3+ oxidation state and that the fraction of Pr^4+^ did not exceed ~20 at.% of the total praseodymium content [44]. Therefore, the contribution of the Pr^3+^ → Pr^4+^ process to the total oxygen uptake can be up to Δδ~0.015. The changes in oxygen content in S85P15 samples on oxidation (and, therefore, the degree or reduction and concentration of Ti^3+^) were found to reasonably increase with the increasing temperature of reductive processing (Figure 9). An exception is S85P15-1700-R-1500 ceramics, which showed an intermediate level of reduction compared to the other samples despite being reduced at 1500 °C. This can be explained by a substantially larger grain size compared to the other samples (Table 2) and dense morphology combined with sluggish oxygen diffusion in donor-doped strontium titanates (as discussed above).

The analysis of the results suggests that the variations in the electrical conductivity of S85P15 cannot be interpreted exclusively in terms of the concentration of electronic charge carriers in the bulk of the ceramics. S85P15-1700-R-1500 ceramics with a moderate degree of reduction exhibit the highest electrical conductivity, while the well-reduced S85P15-H-1350 sample shows the lowest conductivity. This implies that other factors play an important role including microstructural aspects and resistive grain boundaries. An evident distinction of S85P15-1700-R-1500 ceramics from other samples is an order of magnitude larger grain size (Table 2) and, therefore, a comparatively low concentration of grain boundaries. On the contrary, comparatively fine grains in other samples result in a substantial concentration of grain boundaries. It seems that electron scattering on resistive grain boundaries defines the semiconducting behavior and grain-boundary-controlled electrical properties of these samples. This conclusion is supported by the shape of the conductivity curves of S85P15-H ceramics in Arrhenius coordinates which resembles the corresponding *log σ* − 1/*T* dependence reported for reduced nominally undoped SrTiO_3_ [35,55]. Abrantes et al. [55] suggested that this behavior of reduced SrTiO_3_ ceramics can be interpreted in terms of predominant electrical transport along the grain boundaries in the low-temperature range (lower E_A_), while the bulk transport controls the electrical properties at higher temperatures (segment with a higher E_A_). A similar situation is likely to occur in the case of S85P15 ceramics. This is also in agreement with the fact that the activation energy of the electrical conductivity of the S85P15-1350-R-850 sample is close to the corresponding E_A_ values for S85P15-H samples in the low-T range (Figure 8A). The modest reduction of S85P15-1350-R-850 ceramics (Figure 9) should be limited to the surface of grains; the electrical transport in this porous sample (Figure 4B) is controlled, therefore, by the surface layer of the grains and contact points between them (grain boundaries). A lower electrical conductivity in the better-reduced porous S85P15-1350-R-1300 ceramics appears to be caused by the higher porosity (Table 2) and the microstructural aspects—a visibly larger average size of grain/particles (Figure 4D). The combination of these two factors results in a lower actual concentration of contact points between the grains and their agglomerates.

The resistive nature of grain boundaries in donor-doped Sr_1−*x*_La*_x_*TiO_3±δ_ ceramics was earlier pointed out by Moos and Härdtl [37]; they also attributed an apparent transition from high-temperature metallic-like conductivity to low-temperature semiconducting behavior to the grain-boundary-controlled behavior. Comparing the results on the electrical conductivity of A-site-deficient S85L10 and nominally cation-stochiometric S85P15, one may assign a more resistive nature of grain boundaries in the latter material to a specific defect chemistry of donor-doped strontium titanates with the nominal cation-stoichiometric A:B = 1 formulation. As discussed in the Introduction section, donor doping in the Sr_1−*x*_Ln*_x_*TiO_3±δ_ series is compensated by the formation of either A-site vacancies and extended defects such as RP-type SrO shear planes or linear Ln-O_i_ defect clusters. While the extended defects are expected to be located in the bulk of the crystals, thermal treatments in air at temperatures around 1200–1400 °C are often observed to promote the accumulation of such defects at the surface of ceramics in the form of precipitates [56,57,58]. In particular, thermal etching of dense Sr_0.90_Pr_0.10_TiO_3+δ_ ceramics in air at 1400 °C was found to result in the formation of (Sr,Pr)O*_x_* precipitates at the surface [44], probably due to partial exsolution of Sr- and Pr-rich extended defects from the bulk to the surface via grain boundaries. It is likely that nanoscale Sr- or Pr-rich defects are also partly located at the surface of individual grains of as-synthesized S85P15 powder and cannot be completely dissolved into the bulk during the processing under reducing conditions at temperatures ≤ 1500 °C, thus forming resistive grain boundaries. Note that the presence of oxygen excess and, therefore, extended defects in all prepared S85P15 ceramics is confirmed by thermogravimetry (Figure 9). As the oxygen content in oxidized Sr_0.85_Pr_0.15_TiO_3+δ_ should be ≥ 3.075, the TGA results imply that δ is > 0 for all S85P15 samples processed under reducing conditions at different temperatures.

Figure 8B shows variations in electrical conductivity with oxygen partial pressure at 900 °C. The conductivity of comparatively dense S85P15-1700-R-1500 ceramics showed a weak p(O_2_) dependence with only a minor decrease at higher p(O_2_) conditions, apparently due to the sluggish oxygen exchange. Other samples were redox-active and exhibited a decrease in conductivity with increasing p(O_2_) typical for n-type electronic conductors. Assuming a frozen cation sublattice and combining the expression for the equilibrium constant *K*_red_ of the oxygen exchange reaction (Equation (6)) given by Equation (9):

(9) $$K_{red}=\frac{1}{n^{2}\;[V_{O}^{\cdot \cdot }]\;p{\left(O_{2}\right)}^{1/2}},$$

with the definition Equation (8), electrical conductivity is expected to be proportional to ~p(O_2_)^−1/4^ under equilibrium conditions:

(10) $$\sigma =\frac{e\mu_{e}}{K_{red}\;[V_{O}^{\cdot \cdot }]\;}p{\left(O_{2}\right)}^{-1/4}$$

**Figure 10:**
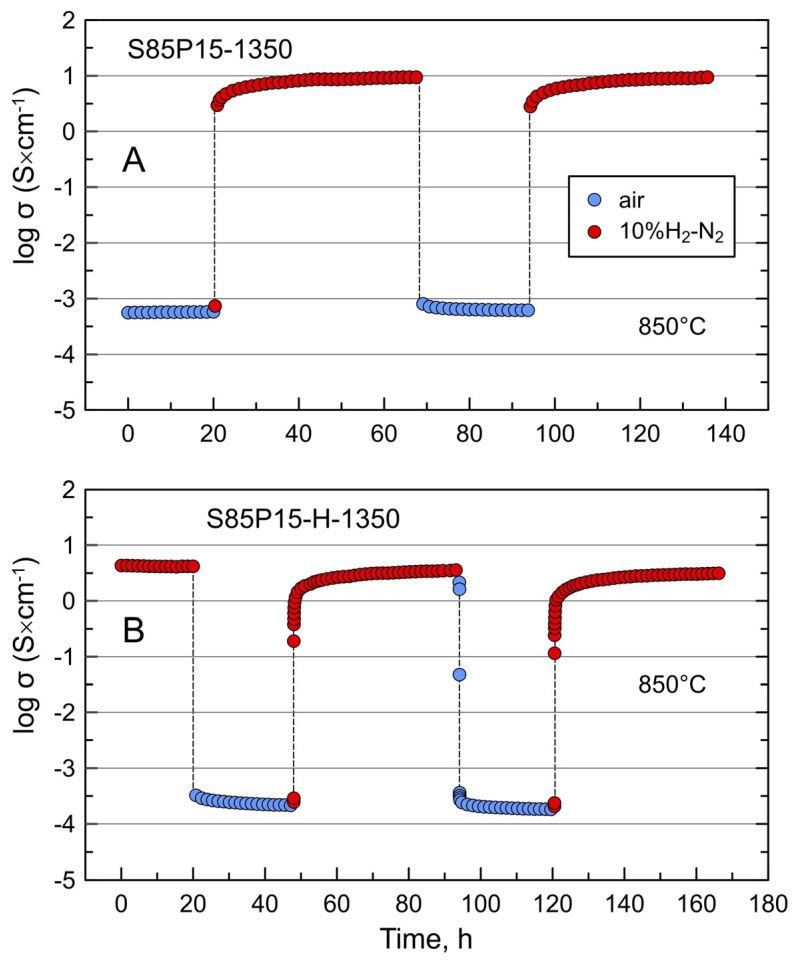
Relaxation of electrical conductivity of porous (**A**) S85P15-1350 and (**B**) S85P15-H-1350 samples on redox cycling at 850 °C. S85P15-1350 denotes the sample sintered in air at 1350 °C for 10 h.

Figure 10 compares the kinetics of conductivity relaxation of two porous S85P15 samples on cycling between oxidizing and reducing conditions at 850 °C. Note that the data were obtained by pseudo-four-probe AC impedance spectroscopy, and the values of conductivity under reducing conditions can be slightly underestimated compared to the data obtained by the four-probe DC technique (Figure 8A) due to non-negligible contact resistance. Contrary to the oxidized S85L10-1320 sample (Figure 7A), oxidized S85P15-1350 ceramics showed a comparatively fast equilibration on switching from an oxidizing to a reducing atmosphere at 850 °C. The conductivity tended to a constant value of ~10 S/cm after 24 h of reduction, and the behavior was reproducible in the repeated redox cycle. Faster reduction kinetics of oxidized “cation-stoichiometric” donor-doped Sr_1−*x*_Ln*_x_*TiO_3±δ_ compared to A-site-deficient Sr_1−1.5x_Ln_x_TiO_3−δ_ at temperatures ≤ 1000 °C were highlighted in the previous works [34,44]. Such behavior reflects an important role of oxygen-rich extended defects in the redox processes in donor-doped strontium titanate at moderate temperatures. Porous S85P15-H-1350 ceramics also showed comparatively fast equilibration on oxidation and re-reduction and a reproducible behavior in the repeated cycles (Figure 10B). The conductivity dropped slightly after the first cycle, but this decline corresponded to ~15%. This suggests that the sample was in a near-equilibrium state after cooling from the reductive processing temperature.

## 4. Conclusions

A-site-deficient Sr_0.85_La_0.10_TiO_3−δ_ and cation-stoichiometric Sr_0.85_Pr_0.15_TiO_3+δ_ ceramics were processed under different thermochemical conditions to produce varying microstructures and levels of reduction. The electrical conductivity of prepared samples was studied under reducing conditions at temperatures ≤ 1000 °C. The results of the analysis of collected data can be summarized as follows:

(i) Reduction of dense donor-doped SrTiO_3_ ceramics is limited by sluggish oxygen diffusion in the crystal lattice even at temperatures as high as 1300 °C. A higher degree of reduction and higher conductivity can be obtained for porous structures under similar thermochemical treatment conditions.
(ii) Metallic-like conductivity of dense reduced Sr_0.85_La_0.10_TiO_3−δ_ is proportional to the concentration of Ti^3+^ and corresponds to the state quenched from the processing temperature. Due to poor oxygen diffusivity in the bulk at temperatures below 1000 °C, the oxygen exchange processes in dense ceramics are essentially limited to the surface. As a result, dense Sr_0.85_La_0.10_TiO_3−δ_ ceramics remain redox inactive and maintain high levels of conductivity under reducing conditions, at least in the short term.
(iii) A similar behavior is characteristic of dense Sr_0.85_Pr_0.15_TiO_3+δ_ ceramics with large grain size (10–40 µm) provided by sintering at temperatures of around 1700 °C. Reducing grain size down to 1–3 µm results in the increasing role of grain boundaries in this material, even for the samples reduced at a high temperature of 1500 °C. Regardless of the degree of reduction, resistive grain boundaries define semiconducting behavior and a lower total electrical conductivity of Sr_0.85_Pr_0.15_TiO_3+δ_ ceramics with fine grain size. The resistive nature of grain boundaries is likely to be associated with the specific defect chemistry of nominally cation-stoichiometric Sr_1−*x*_Ln*_x_*TiO_3±δ_ titanates.
(iv) Oxidized porous Sr_0.85_Pr_0.15_TiO_3+δ_ ceramics exhibit faster kinetics of reduction compared to the Sr_0.85_La_0.10_TiO_3−δ_ counterpart at temperatures below 1000 °C. This underlines the important role of extended defects in the structure of cation-stoichiometric Sr_1−*x*_Ln*_x_*TiO_3±δ_ titanates. On the other hand, the reductive pretreatment of porous Sr_0.85_La_0.10_TiO_3−δ_ ceramics at elevated temperatures (1300 °C) facilitates low-temperature equilibration kinetics on redox cycling.

Thus, titanates of the Sr_1−1.5*x*_Ln*_x_*TiO_3−δ_ series appear to be better suited for the interconnect applications, provided that the ceramics are dense and well-reduced. Sr_1−*x*_Ln*_x_*TiO_3+δ_ titanates are more suitable for the SOFC/SOEC fuel electrodes that are fabricated under oxidizing conditions and then reduced in situ at temperatures close to the operation conditions. However, if the solid oxide cell fabrication procedure allows for the use of pre-reduced Sr_1−1.5*x*_Ln*_x_*TiO_3−δ_ as an electrode component, this may enable a higher conductivity of a porous electrode in combination with adequate equilibration kinetics.

## Figures and Tables

**Table 1:** Literature data on the electrical conductivity of donor-doped Sr_0.90_La_0.10_TiO_3±δ_ ceramics.

Sintering	Reductive Treatment	Measurement Atmosphere	σ, S/cm (900 °C)	Ref.
dry H_2_, 1450 °C		dry H_2_	125	Moos [37]
2%H_2_-N_2_, 1650 °C/8 h		4%H_2_-Ar ^1^	104	Marina [36]
air, 1700 °C/10 h	10%H_2_-N_2_, 1500 °C/10 h	10%H_2_-N_2_	90	Yaremchenko [35]
5%H_2_-Ar, 1500 °C/10 h		humidified 5%H_2_-Ar	87	Li [38]
air, 1450 °C/10 h	H_2_ + 1.2%H_2_O, 900 °C/10 h	H_2_ + 1.2%H_2_O	15	Lv [39]
air, 1650 °C/10 h		humidified 30%H_2_-N_2_	14	Wang [40]
air, 1650 °C/8 h		4%H_2_-Ar ^1^	3.2 ^2^	Marina [36]
air, 1550 °C/50 h		H_2_-Ar ^3^	2.3	Niwa [41]
air, 1400 °C/5 h		30%H_2_-N_2_	1.9	Park [42]
air, 1450–1650 °C/10 h		9%H_2_-N_2_	0.7	Hashimoto [43]

^1^ p(O_2_) = 10^−18^ atm at 1000 °C; ^2^ T = 1000 °C; ^3^ p(O_2_) = 10^−22^ atm at 900 °C.

**Table 2:** Processing conditions and properties of reduced Sr(Ln)TiO_3±δ_ ceramics.

Notation	Sintering	Reduction	Density, g/cm^3^	Relative Density, %	Grain Size, µm
Sr_0.85_La_0.10_TiO_3−δ_					
S85L10-1700-R-1500	air, 1700 °C, 10 h	10%H_2_-N_2_, 1500 °C, 10 h	4.89	96	5–31
S85L10-1700-R-1400	air, 1700 °C, 10 h	10%H_2_-N_2_, 1400 °C, 10 h	4.91	96	5–31
S85L10-1700-R-1300	air, 1700 °C, 10 h	10%H_2_-N_2_, 1300 °C, 10 h	4.88	95	5–31
S85L10-1320-R-1300	air, 1320 °C, 10 h	10%H_2_-N_2_, 1300 °C, 10 h	3.57	70	0.6–6.5
S85L10-H-1450	10%H_2_-N_2_, 1450 °C, 10 h		4.65	91	0.7–6.8
Sr_0.85_Pr_0.15_TiO_3±δ_					
S85P15-1700-R-1500	air, 1700 °C, 10 h	10%H_2_-N_2_, 1500 °C, 10 h	4.80	90	10–38
S85P15-1350-R-850	air, 1350 °C, 10 h	10%H_2_-N_2_, 850 °C, 24 h	3.94	73	0.6–3.0
S85P15-H-1500	10%H_2_-N_2_, 1500 °C, 10 h		4.74	89	0.8–3.5
S85P15-H-1350	10%H_2_-N_2_, 1350 °C, 10 h		3.44	65	0.6–3.2

## Data Availability

Data are contained within the article and available from the corresponding author upon reasonable request.
